# Analysis of marine heatwaves over the Bay of Bengal during 1982–2021

**DOI:** 10.1038/s41598-023-39884-y

**Published:** 2023-08-30

**Authors:** Sudhanshu Kumar, Arun Chakraborty, Raghvendra Chandrakar, Abhishek Kumar, Biplab Sadhukhan, Riyanka Roy Chowdhury

**Affiliations:** 1 Centre for Ocean, River, Atmosphere and Land Sciences (CORAL), Indian Institute of Technology Kharagpur, Kharagpur, 721302 India; 2 Department of Crop, Soil, and Environmental Sciences, Auburn University, Auburn, AL USA; 3 Ocean Process Analysis Laboratory, University of New Hampshire, Durham, NH USA

**Keywords:** Physical oceanography, Climate change, Ocean sciences

## Abstract

Anomalous increase in sea surface temperature and its impact on natural ecosystems greatly interests the research community. Here we investigate the causes, impacts, and trends of marine heat wave (MHW) events in the Bay of Bengal (BoB) from 1982 to 2021. A total of 107 MHW events have been isolated (> 90th percentile threshold) in this Indian Ocean region, and their variation in intensity, duration, and frequency has been investigated. Our research unveils that an average of three MHW events/year accompanied by a linearly increasing trend of 1.11 MHW events/decade has been observed over the study region. It was also found that the most intense event was observed in 2016, which continued for 69 days, and had a maximum intensity of 5.29 °C and a mean intensity of 2.03 °C (above climatology mean). Moreover, it was observed that the net heat flux, along with anticyclonic eddies, was the primary cause of MHW events. Anticyclonic eddies associated with positive sea surface height anomaly were observed (> 0.20 m) in the vicinity of the most intense MHW event. Additionally, climate change and climate modes like El Niño and Indian Ocean Dipole show a high positive influence on the MHW events. Furthermore, we have examined the MHW event recurrence patterns in various regions of the BoB. From the monthly analysis, it was found that August and November had the most occurrences of MHWs, while April and May had the most extreme MHW events.

## Introduction

Since the industrial revolution, there has been a surge in the global average temperature on the Earth’s surface by 1 °C. Anthropogenic activities accelerate this global warming and have led to various extreme climate events, changes in precipitation patterns, sea-level rise, and marine and terrestrial ecological changes. As compared to natural variations, the anthropogenically generated changes in climate have been moving at a much faster pace.

Over the past century, the increase in the mean rate of Greenhouse gases (GHGs) present in the Earth’s atmosphere has been alarming^1^. As per the Intergovernmental Panel on Climate Change (IPCC) report 2013^1^, the projected increase in the global average temperature will be further intensified by such activities, possibly reaching 1.5 °C between 2030 and 2040. It could even approach 3 °C by the end of the twenty-first century, triggering further drastic changes in existing climate patterns.

Over India, the average temperature has risen by 0.7 °C from 1901 to 2018, implying an increase in the sea surface temperature (SST) over the Tropical Indian Ocean. The Indian Ocean is one of the most speedily warming ocean basins in the world^2^, which has warmed by 1 °C alongside a global increase of 0.7 °C.

Oceans have high heat capacity; hence, they absorb nearly 93% of the excess heat due to global warming. This is the major driver behind the increasing trend of SST in global oceans. In the case of the Indian Ocean, 90% of the increase in the SST trend is likely due to anthropogenic emissions^3^. The heat content in the Indian Ocean has also shown a rising trend that is expected to continue^4^.

In the North Indian Ocean, the sea-level rise has shown a considerable increase of 3.3 mm per year since 1993^5^. In addition to changes in sea level and precipitation, climate changes have acted as a catalyst for extreme climate events such as cyclones and heat waves. Due to the Marine Heatwave (MHW) events, the North Indian Ocean breeds more cyclones which also makes the region a regional source of CO_2_ to the atmosphere^6^. The recent decades have shown an increased frequency of cyclonic storms^7^ and extreme temperature events like MHWs^8^.

There are times when SST shows extreme variability locally or globally, which leads to severe stress on the local ecosystem and its associated economies. The study of the variability of SST has a long history in oceanic and climate science^9,10^, and understanding the impacts of heated ocean temperature extremes on marine ecosystems has a key significance^11–15^.

Although an anomalous increase in SST values is well known, over the past century, the frequency and duration of the SST increase have been frequent^16^ and have had huge impacts on marine environments. These extreme events (warm) are responsible for extensive coral bleaching^17^, a decrease in seagrass meadows^18^, and loss of Kelp Forest near the coast of New Zealand and Australia^19,20^ and extensive harmful algal blooms^21^. Moreover, these events have a huge impact on fisheries which are economically important in the northeast Pacific^22^, northwest Atlantic^13^, and coastal Australia^23^.

These extreme, warmer ocean conditions are known as MHWs and are defined as extended anomalously warm ocean conditions above a predefined threshold^24^. A quantitative definition of MHW is a discrete period of the prolonged anomalously warm ocean at a specific location and is based on water temperatures above a fixed^25^, seasonally varying^26^, or cumulative^27^ threshold. An MHW event can last from several days to years. In recent years, MHWs have piqued scientists and the public's interest. As MHW is paired with global warming, ecosystems might have significant impacts.

Atmospheric, land, and oceanic phenomena play significant roles in the occurrence and sustenance of MHW events, with their relative impacts varying with each event. Increased advection, reduced vertical transport, reduced ocean heat loss, increase in solar radiation, stratification, and reduction in mixing, as well as reduced coastal upwelling, could be some of the mechanisms that can generate and control an MHW^28^. The wind speed also reduces when cloud cover reduces due to atmospheric blocking. The increase in the solar radiation received, in turn, increases the SST. If the atmospheric high pressure persists long enough, the warming could lead to the occurrence of an MHW. Therefore, atmospheric preconditioning plays a huge role in the generation of these events.

SSTs are the proxy primarily used to identify MHWs, although MHW may also stretch below the surface^29^. Due to increases in the duration and frequency of MHWs, the number of days these MHWs occur every year has increased globally^30^. With global warming and climate change, this observed tendency is projected to continue^25,31^ alongside the long-term heating of the ocean. In addition, it is suggested that climate change due to anthropogenic activities will increase the possibility of MHWs indefinitely throughout the world ocean in the future^25^, and regionally MHWs shall be driven by unusual weather patterns and disruptions in the ocean currents and mixing. Specifically, a study conducted on data over the period 1925–2016 has shown an increase in MHW duration by 17% and intensity by 34%, which gives us a 54% annual rise in MHW days^16^. Additionally, the sea surface height (SSH) is observed to be influenced by MHWs and is accompanied by an increase in anticyclonic eddies^32^.

In some regions, maximum-intensity MHWs are related to large-scale modes of climate variability. Climate variabilities like El Niño–Southern Oscillation (ENSO), Indian Ocean Dipole (IOD), and more have been found to affect the intensity, duration, and frequency of MHWs. ENSO shows a considerable effect on the generation and modulation of some of the most intense MHWs. During the 2015–2016 El Niño event, approximately three million square km of the ocean surface experienced its highest category of MHWs^33^. In tropical regions, most recognized MHWs have been connected with El Niño events^34^. Being a unique semi-enclosed basin with complicated topography, the Bay of Bengal (BoB) is subjected to different seasonal, interannual, and decadal climatic events, which modulate the upper ocean processes over the region^35–39^.

The understanding of MHW events across the globe is increasing rapidly. However, the studies on MHWs in the North Indian Ocean are very limited, and this paper is an effort to understand and analyze the events in the North Indian Ocean, mainly focusing on the BoB region. Also, it is observed that the rate of warming in this region is much greater than in other regions^40^, an increasing trend in MHWs is observed over the Arabian Sea and the BoB regions^8,39,41^, so it is extremely necessary to understand the patterns of these events. Moreover, a significant increase in duration, frequency, and total days of MHWs was found in the Tropical Indian Ocean from 1982 to 2021^42^. In the northern Indian Ocean, a low production zone, the impact of MHW events can be detrimental to the marine ecosystem as it adversely impacts primary productivity^43^. MHW influences the shift in the phytoplankton community from diatoms to *Noctiluca scintillans*^44^, a decrease in rainfall in the Indian subcontinent^45^, and an increase in flood events in India^46^.

In the Indian Ocean, the positive ENSO phase^47^, Indian Ocean Basin Mode^48^, and IOD mode^49–51^ favor the basin-wise warming of SST. More than 70% of observed heatwave days are influenced by the decaying phase of the El-Niño in the Indian Ocean Basin; however, the weakening of latent heat loss is one of the significant drivers in the origin of most of the MHWs^52^.

There have been several prior studies and published literature relating to the causes and impacts of MHWs in various parts of the world. However, very little research has been carried out on the same in the BoB region despite a significant increase in the frequency and intensity of MHW events since the start of the twenty-first century. This is an important region where detailed research must be carried out. A proper understanding of the genesis of MHW events in the BoB region (4° N−24° N; 76° E−96° E) can help us to predict future MHW events and for planning the required preventive measures. Moreover, heat waves majorly impact various socio-economic sectors, including power and water availability, agricultural production, tourism, health, and many more. More acute and frequent temperature extremes can cause weather-related disorders, directly or indirectly. In this paper, we have tried to describe the way to identify the location of potential sites for MHWs and the characteristics of the longest and strongest MHW events. A month-wise analysis has been explained for the MHWs for the study period (1982–2021). Also, a time-series analysis has been described to find the correlation of MHWs with various other variables. Moreover, an attempt has been made to explain the spatial variation of these MHWs over the BoB region.

## Results and discussions

The BoB is known as the warm tropical ocean basin due to the haline stratification the upper layer of the ocean basin is warmer^53^. In the context of global warming and climate change scenarios, it is much more significant to study the MHW over this region in the recent period. The probable cause and associated impact of these MHW events have been analyzed using different ocean-atmospheric parameters.

**Figure 1:**
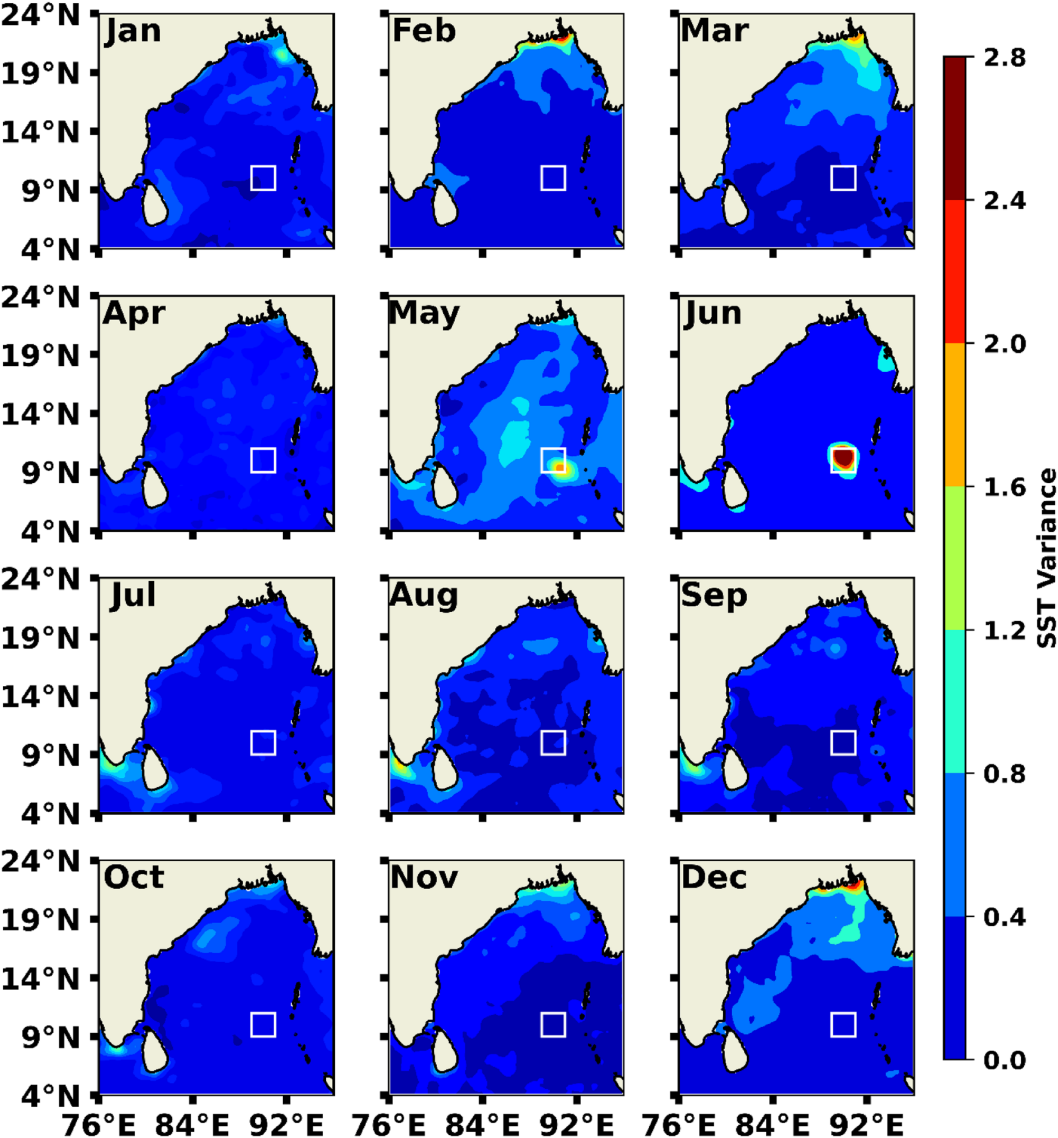
Monthly averaged SST variance of the last 10 years (January 2012–December 2021) for the BoB region (4° N−24° N; 76° E−96° E).

Statistical variance and anomaly of SST is the proxy to detect the MHW events used globally^32,33,54,55^. In this study, we have analyzed the monthly mean SST value variance over the BoB region (Fig. 1) for the recent decades (January 2012–December 2021). A prominent high patch of SST was found near the west of Andaman and Nicobar Islands (9° N−11° N; 89° E−91° E) in May and gradually intensified in June. More than 2 °C SST variance was observed (white box region in Fig. 1) compared to the other region of the BoB. Basin-wide SST variance was comparatively low, having a value of less than 0.8 °C. To understand the probable occurrence of such higher SST variance, we have selected a box region encompassing the high patches (9° N−11° N; 89° E−91° E) for further analysis.

To identify the MHW event over the BoB region, we analyzed the daily SST dataset from 1 January 1982 to 31 December 2021. The criteria (above the 90th percentile threshold) for the identification of MHW have been described in Section “Methodology”.

**Figure 2:**
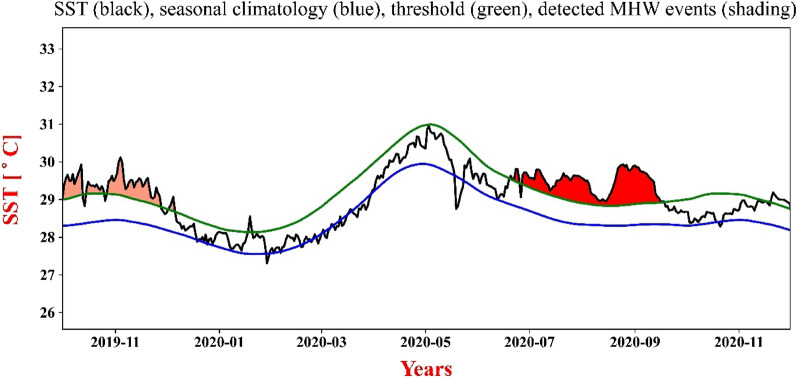
SST (black), seasonal climatology (blue), threshold (green), and MHW event shading of time-series plot for longest duration MHW.

The box region was further examined for MHWs and was observed to have experienced 107 events (of duration 5 days and more), out of which ten events were found to have prolonged for more than 30 days over the study period. The time series of SST, along with the seasonally varying climatology and threshold limit generated, is shown in Fig. 2.

### Occurrence of various MHW events

**Figure 3:**
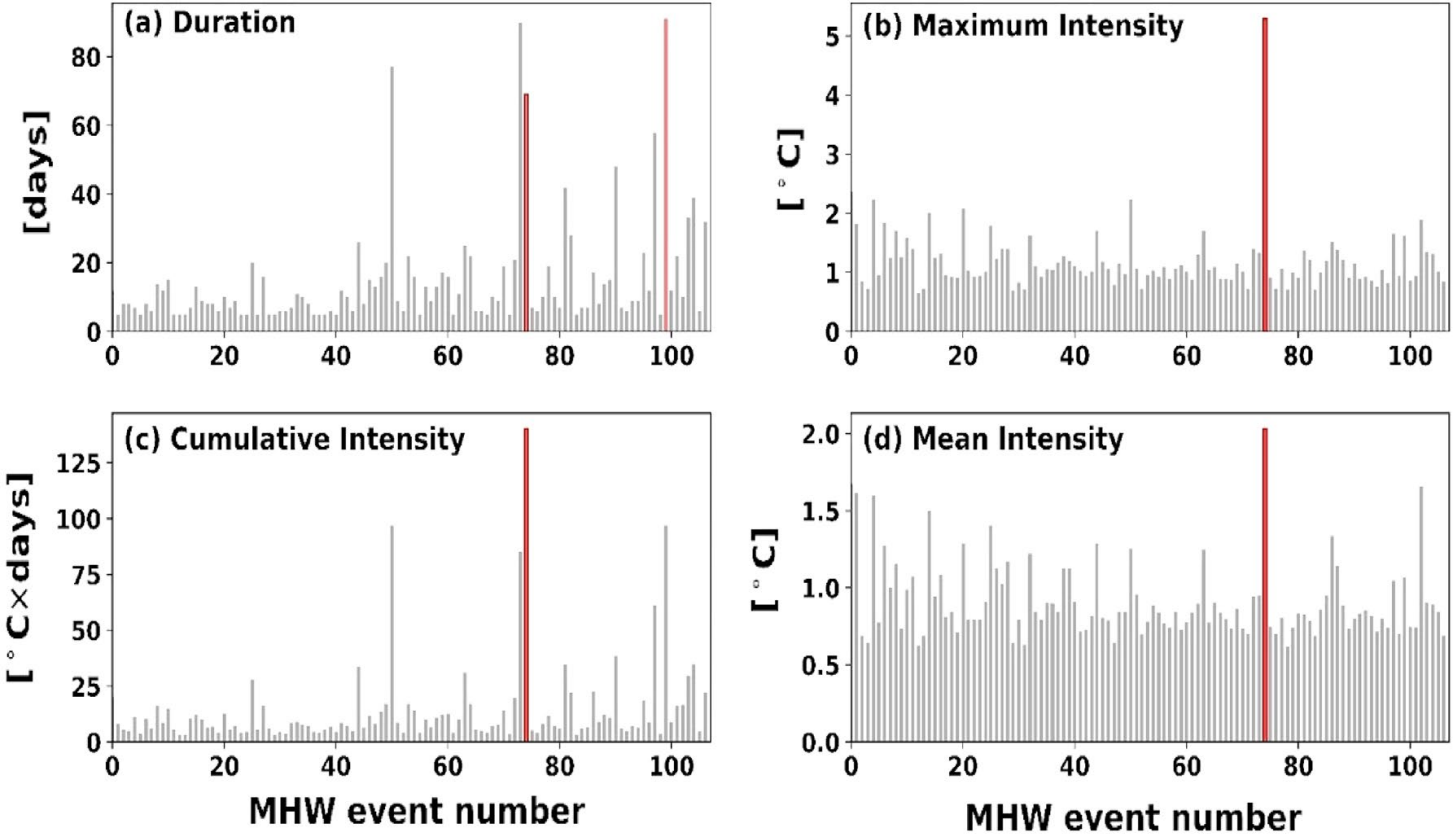
Shows (**a**) duration, (**b**) maximum intensity, (**c**) cumulative intensity, and (**d**) mean intensity of all MHW events that occurred between 01 January 1982 and 30 December 2021. The dark red bar corresponds to the maximum intensity event, and the light red bar is for the event of maximum duration.

As computed, the longest MHW (Fig. 2) lasted for 91 days (20 June 2020 to18 September 2020), having values of 1.62 °C, 1.06 °C, and 96.98 °C-days as maximum (i_max_), mean (i_mean_), and cumulative (i_cum_) intensity respectively above the climatological value (Fig. 3). Cumulative intensity is the product of the mean intensity and duration of a particular MHW event. During this period maximum value of SST was observed at 29.93 °C, while the minimum SST value was 28.92 °C. Although these longer events have moderate intensity, events lasting for a longer duration have implications on marine biodiversity and physical phenomena on a regional scale^34^. Occurrences of the total number of events, their duration, and their intensity (minimum, minimum, and cumulative) have been shown in Fig. 3 during the study period. From our analysis, the BoB region experienced longer MHWs in several years, such as 2010, 2015, 2016, and 2020 which have been prolonged for more than sixty days. Global oceans also have many long-lasting events^56^, and frequent longer MHW events are expected to increase in the future^57,58^. As a result, the risk of MHWs having long-term, severe, and widespread effects on the marine ecology and socio-economic system will rise^19,58^. Hence, a detailed overview of the characteristics and mechanisms of MHWs and associated climatic modes is required, which will aid in the development of high-resolution observation systems and forecasting systems to deal with such extreme MHW events.

**Figure 4:**
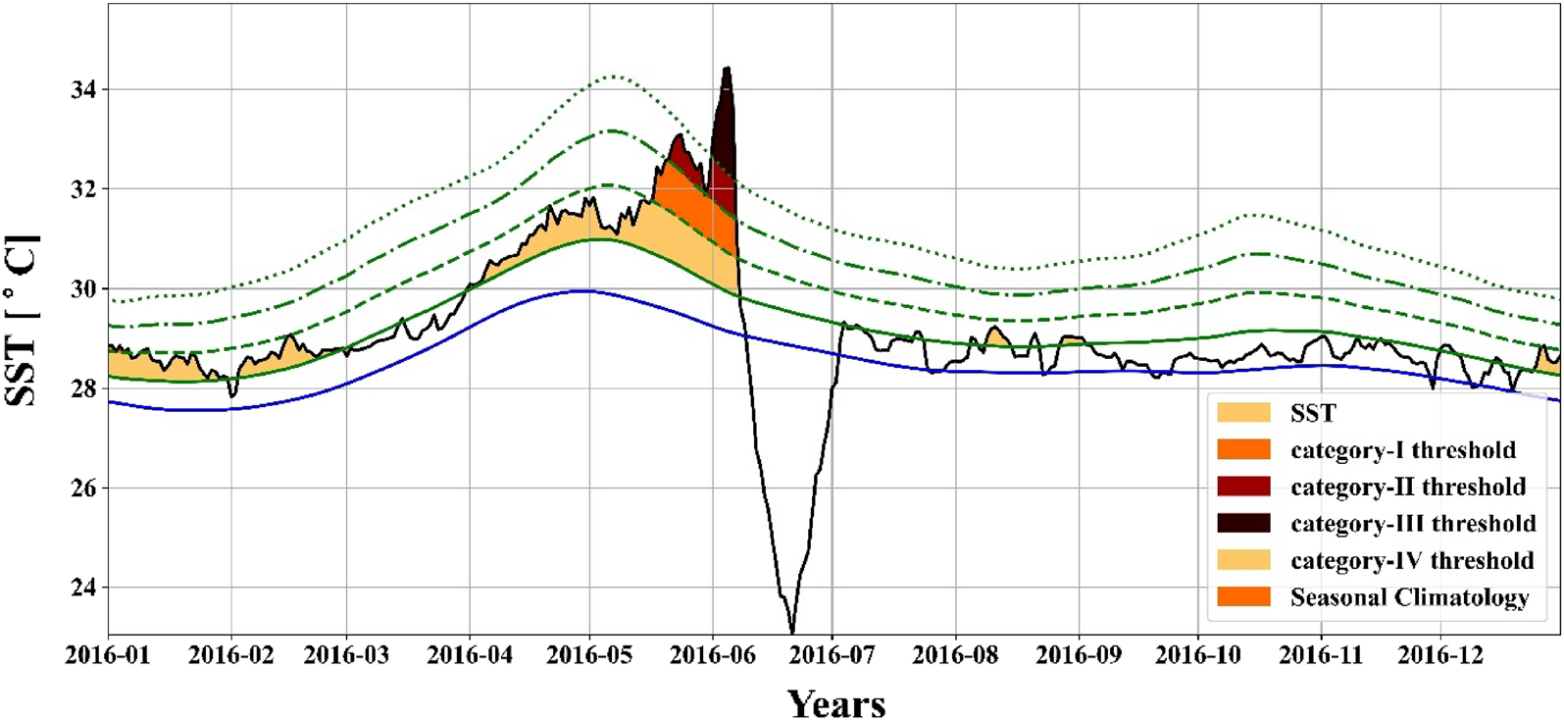
Time series of MHW events with maximum intensity (Red color shades), with different categories by dashed and dotted lines (refer to Table. 1).

The most intense MHW event (Fig. 4) had an i_max_ of 5.29 °C and lasted for 69 days (31 March 2016 to 07 June 2016) with an i_mean_ of 2.03 °C and i_cum_ of 139.93 °C-days (Fig. 3b). This MHW event was the largest for mean and cumulative intensity. It is the fourth-longest duration among MHWs events recorded in the study period. To understand the significance of such MHW, the spatial map of SST for the 69 days long, the 2016 MHW event which started on March 31, 2016, and ended on June 07, 2016, had a maximum intensity of 5.29 °C with a mean intensity of 2.03 °C is presented in the supplementary figures (Figs. S1 and S2). As mentioned in the previous subsection, the box region that has been selected consists of 2° × 2° grid size; however, this event contains an extreme category MHW event (Table 1). This event of 69 days with a maximum intensity of 5.29 °C had 7% of days in the extreme category. Furthermore, the event had 16%, 9%, and 68% as severe, strong, and moderate categories, respectively. As compared to moderate and strong MHWs, severe (Category III) and excessive (Category IV) MHWs have more profound biological effects^30^. Because the thermal maximum of essential habitat-forming species exceeds during an extreme MHW, it significantly impacts entire temperate populations, resulting in wide-ranging cascade impacts^19^.

**Table 1:** Categorization of MHW events based on the intensity at each point in space and time.

Category	Type	Magnitude
I	Moderate	1–2 × D
II	Strong	2–3 × D
III	Severe	3–4 × D
IV	Extreme	> 4 × D

D is the difference between the climatological mean and the 90th-percentile threshold.

With the projected increase in SSTs in the future, additional categories V or VI, may be observed, which will be detrimental to the marine ecosystem. Since within the same MHW category, the impacts were still varied, and the event indicates the requirement of consideration of thermal tolerances of affected organisms and hydrodynamic conditions. Moreover, future policy decisions should examine the effects of long-term temperature fluctuations and these short, acute episodes, which have profound consequences long after they have passed.

**Figure 5:**
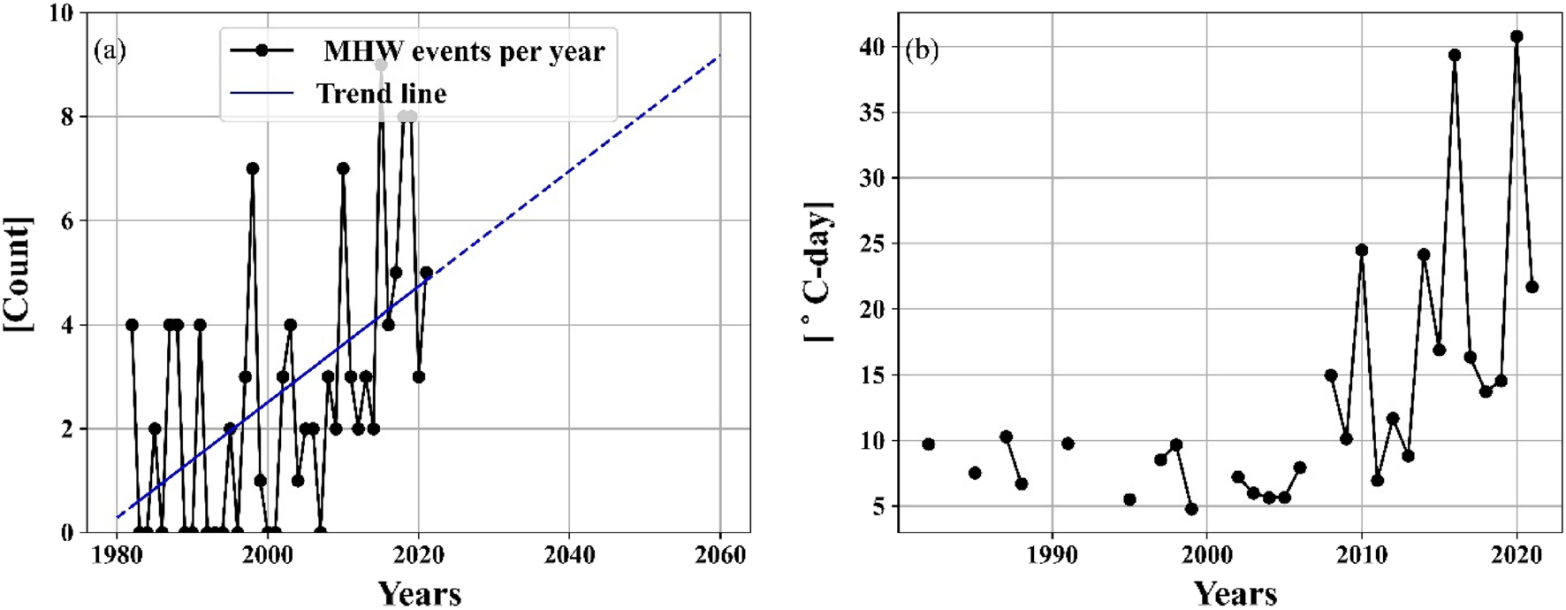
Time series of (**a**) number of MHW events per year from January 1982 to December 2021 (black) and trend line (blue), (**b**) year-wise average MHW cumulative intensity.

The annual MHW frequency trend from 1982 to 2021 is shown in Fig. 5 for the region. It was also observed that there were, on average, 2.675 MHWs events each year, with a linear upward trend of 1.11 MHW events per decade (Fig. 5). This increasing trend is statistically significant (refer to^52^ for details). It can also be noted that during the last 10 years, more than 5 MHW events occurred every year compared to only 2–3 events before 2010.

On projecting this, linear trendline for the next few decades, it shows substantial upward trends in the yearly cumulative intensity and frequency, which gives a worrisome picture. If these trends persist, the BoB will experience heatwaves every month in the next 55–60 years, and it will attain a semi-permanent heatwave state by the end of the century, with extreme temperatures present for more than half of the year. The average cumulative intensity is 1.21 °C-days per year. It can be observed that the trend is increasing at a very fast rate, and for the past decade, the average cumulative intensity was around 20 °C-days per year. These would have a very devastating impact on the regional ecosystem, aggravating the effects of pollution of nutrients, increasing the severity of low-oxygen “dead-zones”^59,60^, stimulating algal blooms, stressing or killing bottom-dwelling communities, causing shifts in species composition^61^, and leading to declines in important commercial fishery species^41,62^.

**Figure 6:**
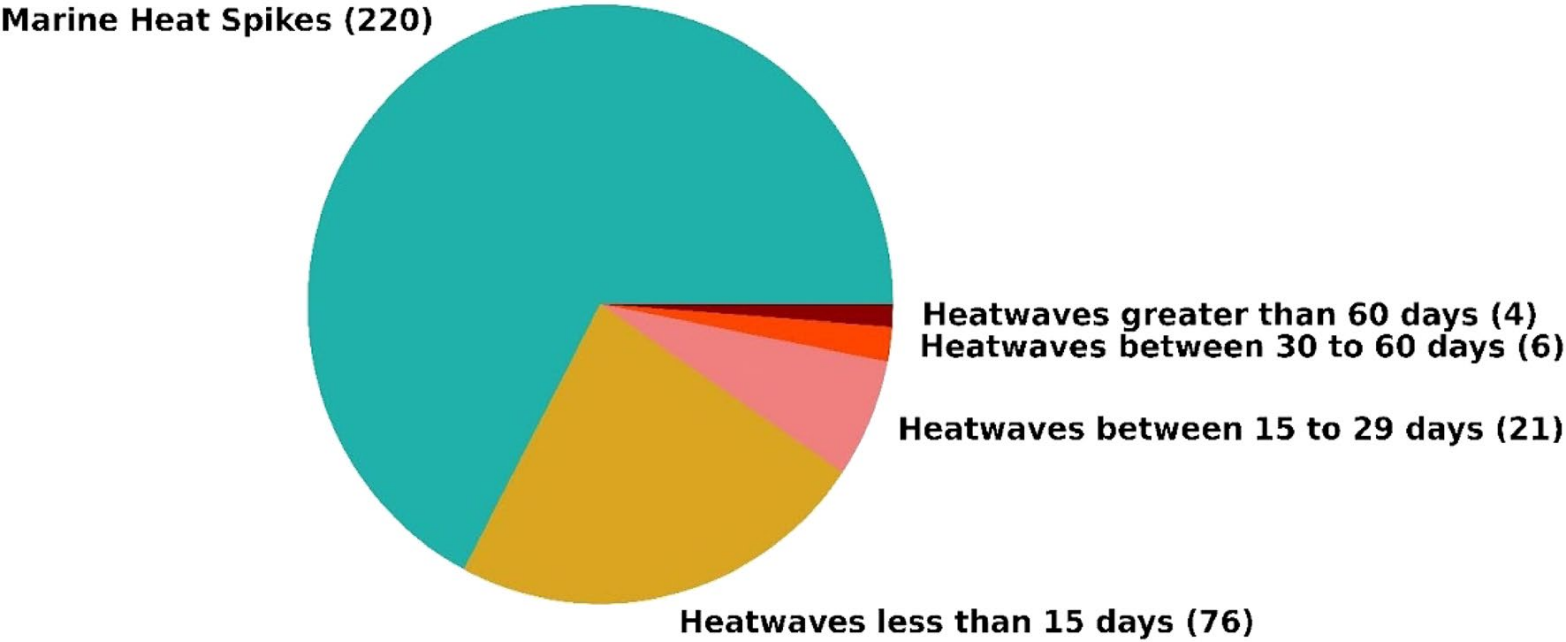
The categorization of duration in the number of days heatwaves or heat spikes lasted at the selected location.

Upon investigating MHSs in the study region, the same has been plotted in Fig. 6. It was observed that 220 MHSs lasted from one to four days. The most intense MHS had a maximum intensity of 1.75 and an average intensity of 1.54 °C above the climatological mean. The MHW events and cumulative intensity show an increasing trend (Fig. 5), indicating that the increase of the larger and more intense heatwave events will be observed in future decades with climate change-related temperature increases^57,58^. It will also influence MHSs because these observed spikes are also expected to last for a longer duration with increased intensity and will be considered an MHW event if it lasts 5 days or more.

### Month-wise analysis of MHW events

**Figure 7:**
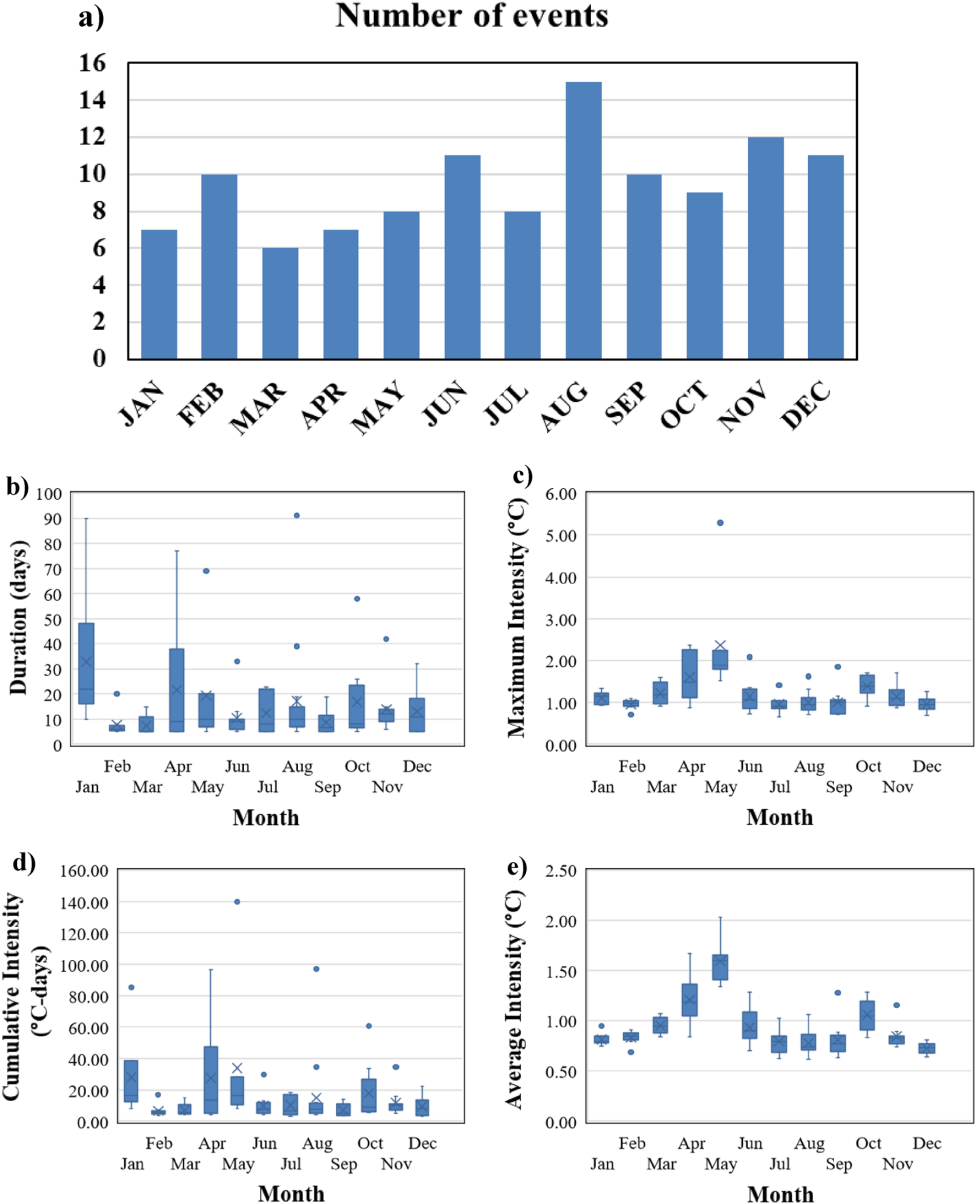
(**a**) Monthly bars for the number of MHW events, (**b**) duration, (**c**) maximum intensity, (**d**) cumulative intensity and (**e**) mean intensity are shown using boxplots from 1982 to 2021 for MHW events.

The monthly variation of MHW frequency from 1982 to 2021 shows maximum events during August followed by November, December, June, and February compared to other months (Fig. 7a). This MHW frequency analysis for the months is consistent with the previous study^41^. It is also observed that the duration and cumulative intensity of MHW events in January and April have a larger mean than in other months (Fig. 7b,d). The biannual signal is observed for maximum intensity and average intensity MHW events with the highest intensity peaks during May and October (Fig. 7c,e).

### Reasons for the occurrence of MHW event

#### Net heat flux and MHW generation

**Figure 8:**
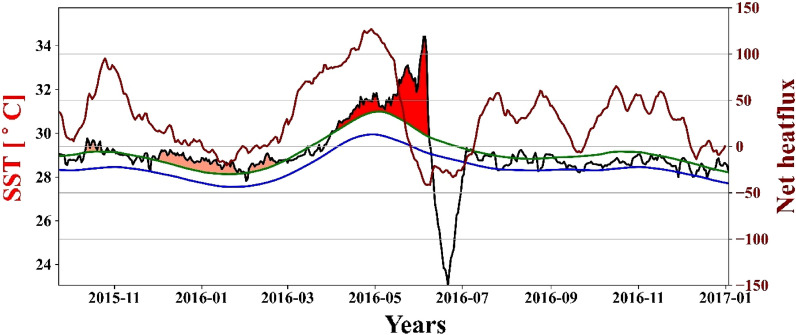
Temporal variation of SST (black), seasonal climatology (blue), threshold (green), and MHW event shading in time series from October 2015 to December 2016 and net heat flux (dark red) for the same period.

The temporal variation of SST, its climatology, the 90th percentile of SST, and net heat flux from October 2015 to December 2016 are shown in Fig. 8. It can be observed that just before (some lag) each MHW event, the net heat flux has increased in its value, which means there would be more heating of sea surface water and the cause of the MHW event^41^. The recent study^42^ also suggests that these heat fluxes and anomalies play a very important role in MHWs in the tropical Indian Ocean (TIO) region. Moreover, Strong air-sea heat flux anomalies are related to diverse climate modes in the TIO, implying that atmospheric forcing may also play an important role in MHWs. Therefore, the increase of net heat flux before the heat wave events is one of the factors for the initiation of the MHW.

#### Surface ocean current (SOC) for MHW generation

**Figure 9:**
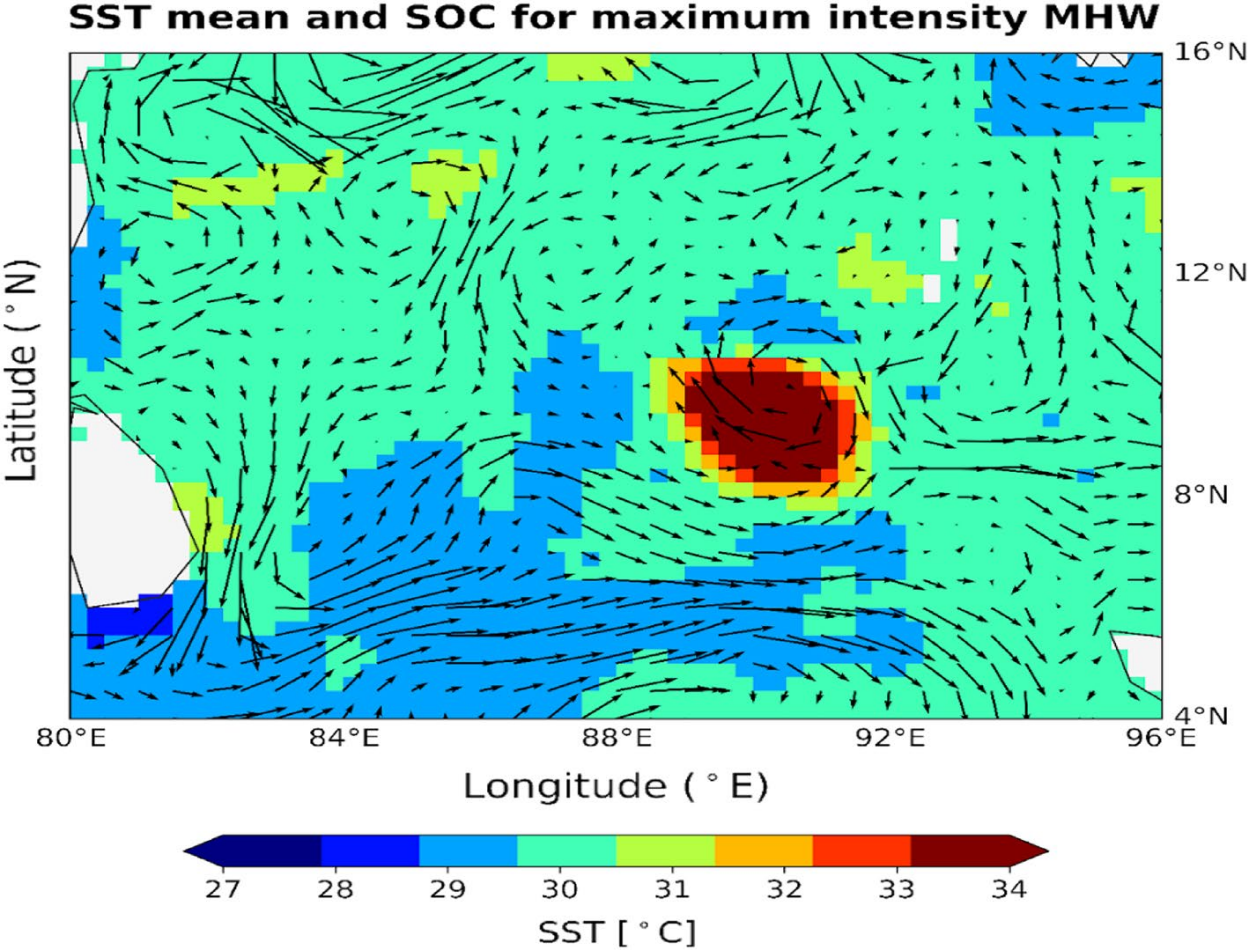
Spatial variation of SST (color) and SOC (vector) for the most intense MHW event.

The average values of SOC and SST were calculated and plotted for the most intense (Fig. 9) and longest MHW event duration (not shown). It is observed that the currents flowing around the study area of the BoB region are forming a clockwise pattern leading to downwelling and suppressing the cold water to come above to cool the region. The surface currents are weak in the surrounding area, and that will diminish the advection of waters from the nearby regions. All these lead to a higher heat concentration in the area as hot water is very slowly being advected, and it creates a favorable condition for MHW events to occur. It can be reported that the weak surface ocean current is one of the supporting causes of the MHWs in the study region, as ocean advection influences their formation and decline^63^.

#### MHWs and change in sea surface height

**Figure 10:**
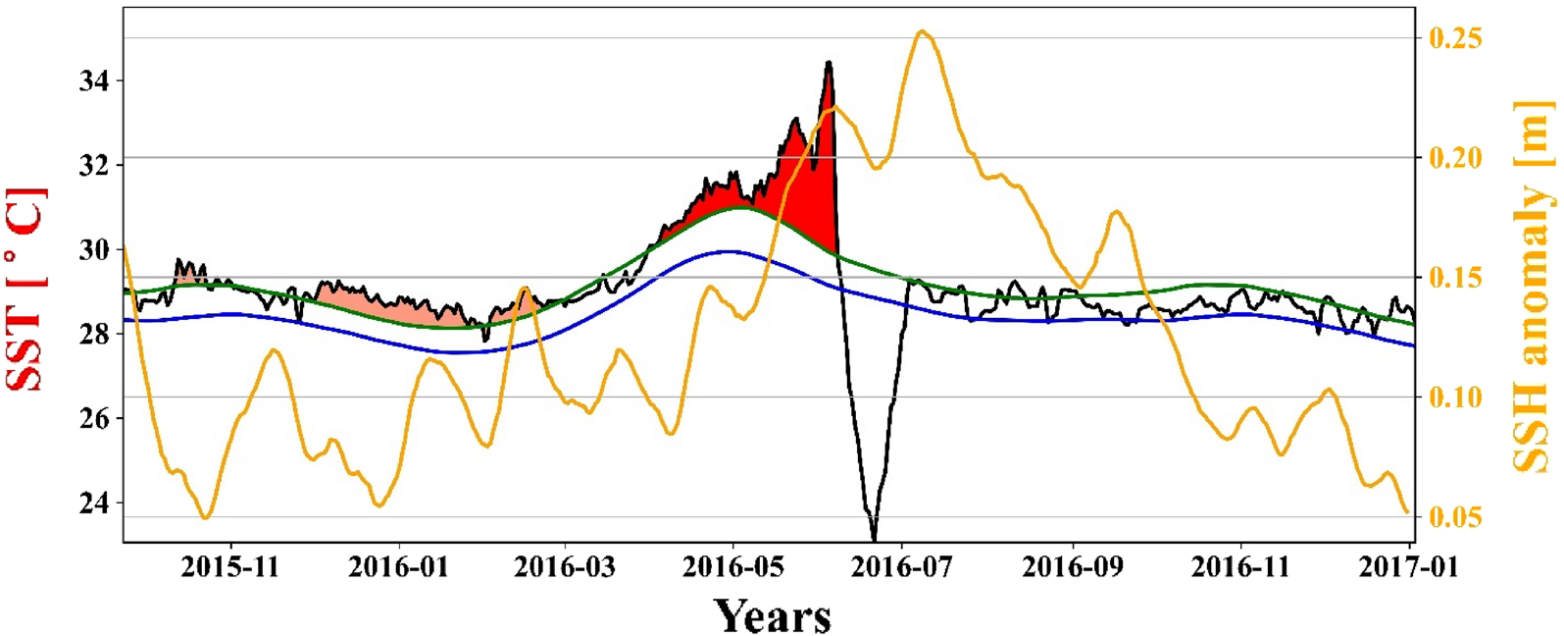
Temporal variation of SST (black), climatology (blue), threshold (green), and MHW event shading in time series from October 2015 to December 2016 and SSH anomaly (Yellow).

On plotting the time series of the study area average values of SSH anomaly and SST for the most intense (Fig. 10) and longest MHW event duration (not shown), it was observed that the anomaly follows the MHWs with a lag of around 20–22 days. Whenever an MHW event occurs, it leads to an increase in SSH, and a positive spike in SSH anomaly is observed; for instance, during the most intense MHW event, SSH anomaly increased to 0.25 m from a mean value of 0.07 m, but the correlation is not strong and may be due to location-dependency^64^.

Moreover, several previous researches have suggested that the ocean mean state could modify the efficiency of oceanic wave-induced upper-ocean mixing, altering the MHWs^65,66^. Moreover, the duration of thermocline warming generated by oceanic waves is a key element in maintaining MHWs^67^. This is one of the possible explanations for significant SSH anomalies.

#### MHWs and change in E-P

**Figure 11:**
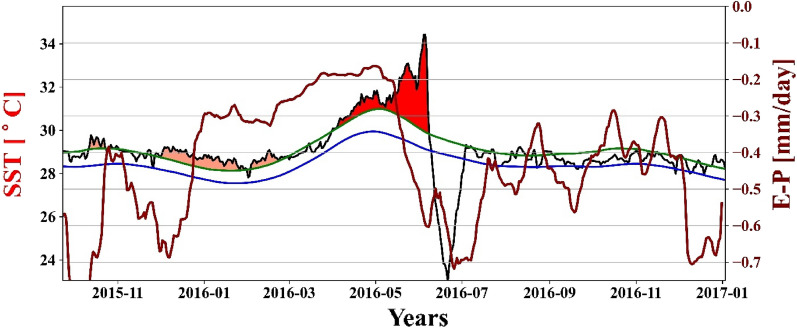
Temporal variation of SST (black), seasonal climatology (blue), threshold (green), and MHW event shading in time series from October 2015 to December 2016 and the difference between evaporation and total precipitation (dark red) for the same period.

On observing the correlation between heating events and E−P values, it was found that the E−P value increases with an MHW event and leads by 20–25 days for intense events; however, the lead period is lesser for low-intensity MHW events (Fig. 11).

This change in the lead period is also due to the occurrence of MHW events during the summer and winter months^63^. As stated above, net heat flux, which is the driver of MHW, is influencing the E-P values, and a positive correlation with MHWs is observed. The results are supported by^63^, as they state that Air-sea heat flux alone may not be the only factor influencing the genesis of MHWs.

#### Influence of ENSO and IOD on MHWs

**Figure 12:**
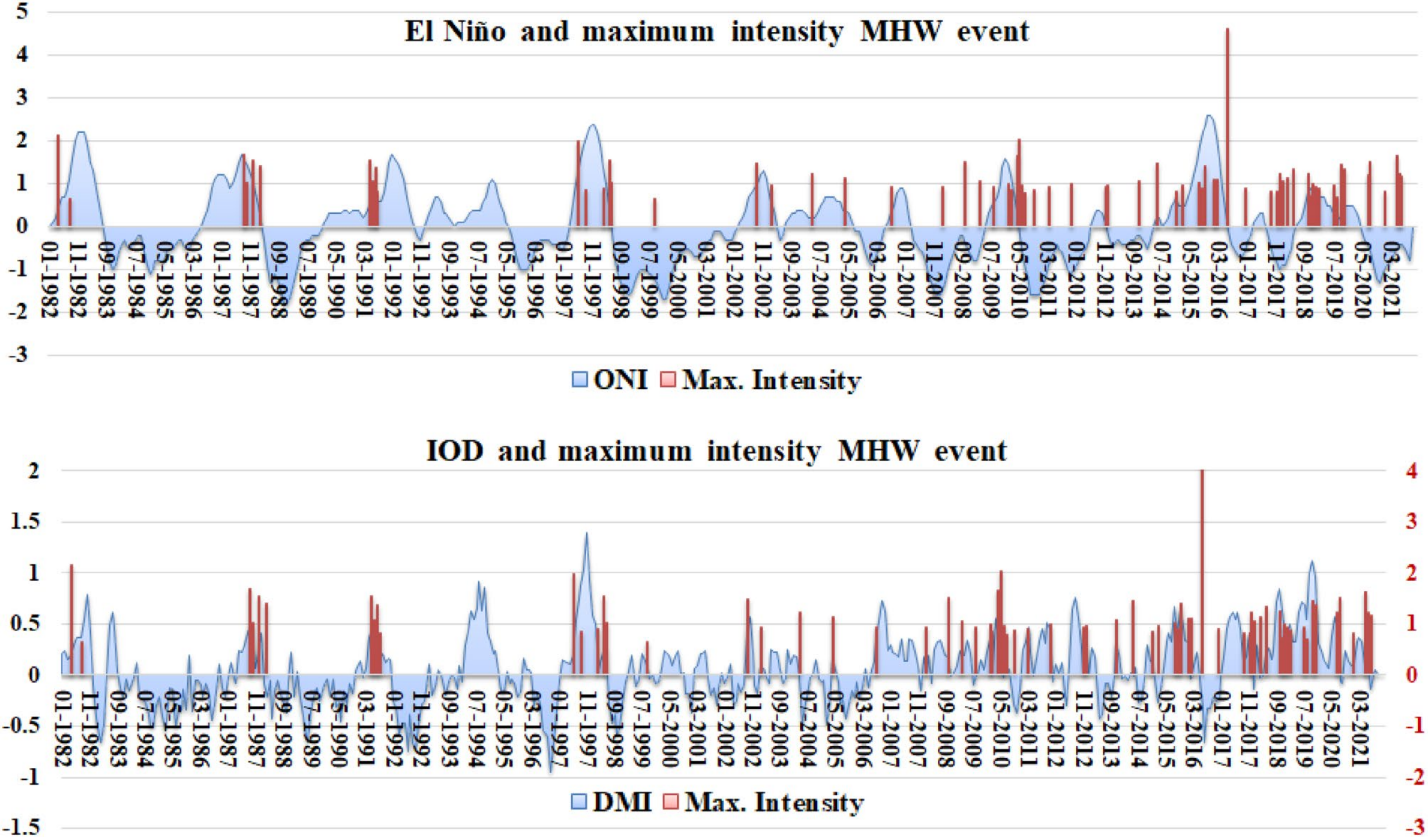
Time series for ONI (top) and DMI (bottom) with the maximum intensity of MHW events for the period 1982 to 2021.

Two important climate modes, ENSO and IOD, were studied and plotted with the maximum intensity of all MHW events between 1982 to 2021 (Fig. 12). Both ONI and DMI show a positive correlation with MHWs for the study area. This confirms the warming of the BoB by ENSO and IOD. In general, most MHWs occurred during strong El Niño till 2000. However, after 2000, MHW events were observed during negative Ocean Niño Index (ONI) values. A similar trend is observed for the Dipole mode Index (DMI) with MHWs.

During the positive IOD phase, there is a warming of the BoB and most of the MHW events coincide with the positive IOD phase. The positive phase leads to the formation of the BoB Warm pool. The Indian Ocean Basin Mode clearly affects the duration of MHW days^57^, with a significant common period when the basin-wide warming peaks. An indirect effect of ENSO on the Indian Ocean is also observed as the Indian Ocean undergoes basin-wide warming following an El Niño event.

A very important pattern is observed here. Before 2000, the influence of climate variabilities was dominant over that of warming. However, after 2000, the dominance of warming in the generation of MHWs is of great importance. Figure 12 show that warming due to anthropogenic climate change has substantially affected natural climate variabilities since the 2000s in the generation of MHWs, and it further increased after 2010.

MHW occurrences in northern Australia during 2015–2016 coexisted with strong El Niño that dissipated in austral autumn 2016, negative IOD, and a low Australian monsoon index^68^. Benthuysen et al.^68^ believe the atmospheric heat transfer was especially strong during the 2016 MHWs, warming the upper ocean significantly. El Niño creates regional warming patterns across Australia as a result of changes in the results, which are in good agreement with previous findings (e.g.,^16,68^), and it is expected that this study will be a first step toward planning the future of MHWs observation.

### Spatial variation of MHW events in the BoB region

**Figure 13:**
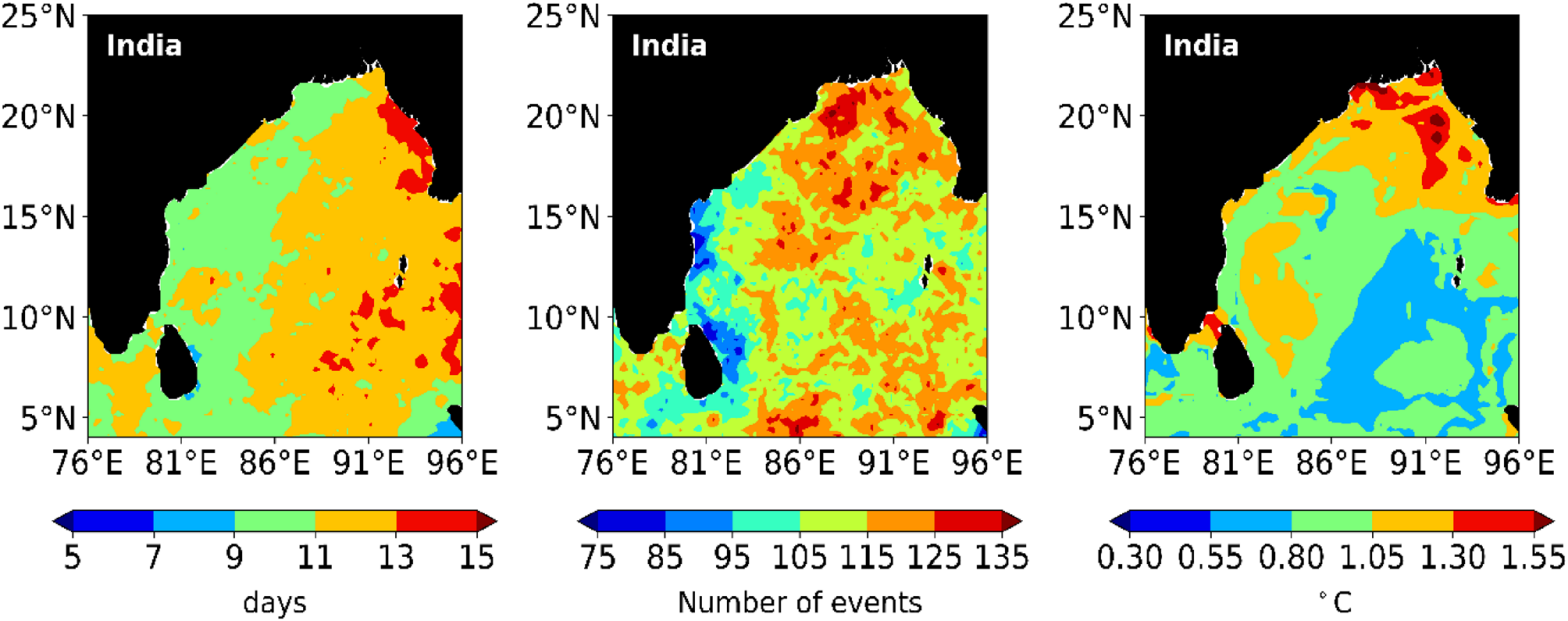
Spatial variation for the occurrence of average duration of MHWs per year (left), the average number of MHW events per year (center), and annual mean intensity of MHW events (right) in the BoB region for the period (1982–2021).

The MHW duration annual mean plot (Fig. 13) shows a clear increase of about 3–5 days per year between the western and eastern BoB regions. These regions in the western and eastern BoB show a trend of around 9 and 13 days, respectively, of MHW per year. At some locations near the Andaman and Nicobar Islands, as well as Myanmar, the duration ranges from 13 to 15 days per year. From the southern to the northern part of BoB, at the same longitude does not show any significant change in the trend during this period. The total MHW events varied across the BoB (Fig. 13). MHWs are ubiquitous in the study region and range from 70 to 140 in 40 years period, depending on location. The longest duration of over 115 events is distributed sporadically in the northern part, while other regions are most commonly characterized by events of 95–105 days, except for near Andhra Pradesh state of India and the eastern part of Sri Lanka, which has fewer than 85 MHW events. MHWs frequency is likely to increase all over the BoB^42^. Regarding the annual mean intensity of MHWs (Fig. 13), in the northern region and the Palk Strait region, there is a mean of 1.25–1.5 °C per year or even higher at some locations. In the southern and western BoB, the mean intensity is about 0.5–1.0 °C per year. These regions have low-intensity waves, but the duration and frequency are more.

Hence, in the long term, while the MHW count has remained more or less the same over the region, the annual mean intensity has increased by nearly 1.5 °C. Both the duration of the event and intensity are higher in the northern BoB relative to other parts. Therefore, prolonged and intense heat waves are observed. Moreover, along the entire length of the BoB, MHWs tend to occur at nearly the same time. In this region, the intensity of MHWs is also predicted to increase^69^.

## Conclusions and future scopes

The main focus of this study was the BoB (4° N−24° N; 76° E−96° E) of the Northern Indian Ocean and to find maximum contrast in SST variance as locations having high contrast will have high chances of having MHW events. These events are generally abnormal SST changes from the mean temperature for a prolonged period. From observations, it was found that 9° N−11° N; 89° E−91° E near the Andaman and Nicobar Islands is showing the potential site for longer and more intense MHW events. So, the area with the highest variance was chosen for analysis, and it was concluded that more than 107 MHW events occurred at the selected location for the study period from 1982 to 2021.

Upon analysis, it was found that the longest heatwave of 91 days and the most intense heatwave had a maximum intensity of 5.29 °C above seasonal climatology lasting for 69 days which ranks fourth in the duration of all MHW events. This most intense heatwave falls under category IV (refer to Table 1 for categorization) and had detrimental impacts on the ecosystem. Moreover, on further analysis, a linear increasing trend of frequency of 1.11 MHW events per decade was obtained, which is statistically significant. On forecasting this trendline, we could comment on the large increase in the frequency of heatwaves in upcoming decades having an impact on various other variables. Furthermore, in monthly analysis, it was successfully observed that maximum heatwaves occur in August, followed by November, and most intense events occur in April and May.

There are two main reasons for the occurrence of MHW, the first is net heat flux and the second is ocean currents pattern by making the SST rise through the advection of warm water at the ocean surface. IOD and ENSO also influence the occurrence of MHW events. More significantly, a very clear relationship between these variables and MHWs was established.

It was also seen that, over the past few decades, MHW generation in the BoB is dominated by ocean warming due to anthropogenic climate change as opposed to natural climate variability. Thus, it can be said that, in recent years, SST trends have driven MHWs in the BoB. This could mean an increase in the duration and intensity of these events as warming increases.

From all this, it can be concluded that the biggest MHW event of the year 2016 in the Northern Indian Ocean region was caused by the effect of increased SST caused by global warming. The time series of MHW events were explored for finding a correlation. The positive correlation of MHWs with SSH anomaly and E-P was also observed. The spatial plot gave more insightful information about various regions of BoB where MHW has relatively more impact. Frequent and more intense MHW events were observed in the northern part and longer ones in the eastern part of BoB.

In the future, these events and their drivers could be explored in detail, and their impacts on the ecosystem could be studied in the Indian Ocean. The relationship between warming events such as MHWs and an increase in the occurrences of cyclones in the Indian Ocean^70^ in recent years can be investigated. Using the latest development in machine learning techniques, the future predictions for MHW events could be applied to the Indian Ocean similar to various regions around the world oceans. Furthermore, the MHWs have a huge impact on regional biogeochemistry^8^, may alter dissolved oxygen, and may act as a catalyst for various acidification reactions. In future studies, these can be explored using numerical/coupled modeling.

Organisms may adapt to climate change to some degree^71^, but the rate of adaptation varies greatly between species^72,73^, and differences in the extent to which marine species must adapt due to geographic variability in SST variations are possible. As a result, the outcome has far-reaching ecological, social, and economic consequences, necessitating a response strategy from affected communities and policymakers.

## Data and methodology

Different datasets may provide different heatwave information despite using the same metrics due to a change in the dataset's resolution, quality, consistency, or instrumentation concerns. Datasets with a high spatial and temporal resolution exhibit more variability than datasets aggregated over larger areas or based on longer time means (smoother)^73^. Not only could various datasets provide different answers for the same metric, but also, some indices may be unacceptable or impossible to derive from specific data sources^74^. So, it is recommended that when calculating MHWs, the highest quality available dataset should be used. In this research work, the following datasets have been used.

### Datasets used

#### Sea surface temperature (SST)

SST data used was obtained from Daily Optimum Interpolation SST version 2 (OISST v.2), which has a daily temporal and horizontal resolution of 1/4° × 1/4° grid available at the National Climatic Data Center (NCDC) of the National Oceanic Atmospheric Administration (NOAA)^75^. For this research work, daily SST datasets were analyzed for the period of 1 January 1982 to 31 December 2021 unless stated otherwise for the identification of MHWs and comparison with other variables. The datasets are available at https://psl.noaa.gov/data/gridded/data.noaa.oisst.v2.highres.html.

#### Air-sea heat flux

The air-sea fluxes are extracted from the TropFlux reanalysis product with a horizontal resolution of 1° × 1° over the BoB region^76,77^. The turbulent fluxes were calculated using the modified daily averaged input parameters with the Coupled Ocean–Atmosphere Response Experiment (COARE) version 3.0 algorithm^78^. The datasets used are available at https://apps.ecmwf.int/datasets/data/interim-full-daily/levtype=sfc/.

#### Sea level anomaly (SLA)

Altimeter-based merged satellite SLA is computed on a twenty-year (1993–2012) climatology. The SLA is estimated based on the Optimal Interpolation and measurements merged from the different altimeter missions available (see http://duacs.cls.fr for processing details)^79^. Finally, merging all the flying satellites, an Optimal Interpolation is made to compute gridded SLA. The datasets used are available at https://resources.marine.copernicus.eu/products.

#### Surface ocean current

Surface ocean current data having a horizontal resolution of 0.25° on a uniform latitude/longitude projection^80^ is downloaded from the global ocean physics analysis and forecast product provided by Copernicus, which are weekly coupled atmosphere–ocean data assimilation and forecast system. For the atmospheric configuration (~ 40 km resolution) Met Office Unified Model v10.6 is used, which is coupled with the oceanic configuration taken from the hourly NEMO v3.4 and the multi-thickness-category sea ice model CICE v4.1 (both on the ORCA025 grid) has been used by the system. The datasets used are available at https://resources.marine.copernicus.eu/products.

#### Evaporation and total precipitation

These datasets are downloaded from ERA5, which is daily updated with a latency of nearly 5 days^81^. For the reanalysis, data were gridded to a regular grid size of 0.25° and 0.5° for the uncertainty estimate (0.5° and 1°, respectively, for ocean waves). Negative and positive values indicate evaporation and precipitation, respectively, with a convention that downward fluxes are positive. The available hourly datasets were converted to the daily mean for analysis purposes. The datasets used are available at https://cds.climate.copernicus.eu/cdsapp#!/dataset/reanalysis-era5-single-levels?tab=form.

#### Oceanic Niño index (ONI)

The monthly data of Niño 3.4 (5° S–5° N and 170° W–120° W) over the Pacific Ocean from the year 1982 to the year 2021 is used. Furthermore, throughout 40 years, data of the three-month moving average anomaly of the Niño 3.4 region, ONI. According to ERSST.v4, the base period for this three-month rolling mean of Niño 3.4 SST anomalies is 1971–2000^82^. These data sets used available on the Climate Prediction Centre website (http://www.cpc.ncep.noaa.gov/products/monitoring_data/).

#### Indian ocean dipole (IOD) index

The strength of the IOD is represented by an anomalous SST gradient between the western equatorial Indian Ocean (50° E−70° E; 10° S−10° N) and the southeastern equatorial Indian Ocean (90° E−110° E; 10° S−0° N). Dipole Mode Index is the name given to this gradient (DMI). The phenomenon is known as the positive (negative) IOD when the DMI is positive (negative). It is calculated using the HadISST1.1 SST dataset for the entire period^83^. Climatology is currently from 1981 to 2010. The monthly data from 1982 to 2021 has been used, which is periodically updated from the NOAA website https://psl.noaa.gov/gcos_wgsp/Timeseries/Data/dmi.had.long.data.

### Methodology

In this research, for determining an MHW event, the temperature has to surpass an upper locally determined threshold (above the 90th percentile relative to the long-term local climatology) which permits variability occurring due to regional differences as opposed to an absolute temperature threshold. The event needs to persist for more than 4 days to be considered an MHW, thus making it a “prolonged” warm water event. MHWs also have well-defined start and end times and are termed “discrete”. However, this definition of MHWs allows gaps of two days or less between heatwave events.

For climatology, a thirty-year baseline period of 1982–2011 (inclusive) was used in our study to estimate the mean climatological SST and percentile thresholds. The climatology is calculated over a selected baseline period, ideally a thirty-year temperature data^84^. Hobday et al.^26^ adopted a daily local upper-percentile climatology as the threshold at which MHWs are detected, consistent with atmospheric heatwave criteria^85^. As the anomaly of warm ocean temperature surpasses the threshold for shorter than five days, it is categorized as a “marine heat spike (MHS)” and is not recognized as an MHW.

To understand the MHW characteristics, three parameters were observed: MHW Duration, i.e., the number of days of the event, MHW frequency, i.e., the number of events; and MHW intensity, i.e., the maximum anomaly of the event that occurred in a season or year. All assumptions used are the same as Hobday et al.^26^.

MHWs have been categorized based on their characteristics on a scale from category I to IV, as proposed earlier by Hobday et al.^30^. This anomaly differs with the time of year and location^26^.

For a detailed monthly analysis, all MHW events were further divided into monthly events for the period 1982 to 2021 for further variation analysis. It is to be noted that some events started in a particular month and ended in the next month, in those cases considered to be an event of the month in which it occurred for a larger duration. A few events lasted for two months and more and were considered events for both months for monthly trend analysis. Furthermore, the causes and impacts of MHWs on various physical variables have been analyzed, which are SSH, heat flux, surface ocean currents, and the difference between evaporation and precipitation. At last, how MHW events vary in different regions of BoB has been broadly analyzed.

This study is limited to detecting MHWs using satellite data only due to the lack of in situ measurements. Several previous research has used SST from satellite products to identify MHWs and draw broad inferences based on those few direct temperature data^32,86,87^.

### Supplementary Information

Supplementary Figures.

## Data Availability

All the data used in the study were downloaded from the open source and the website details are given under Data and Methodology section. Moreover, the datasets/codes used and/or analyzed during the current study are available from the corresponding author upon reasonable request.
