# Author Correction: Analysis of marine heatwaves over the Bay of Bengal during 1982–2021

**DOI:** 10.1038/s41598-023-43101-1

**Published:** 2023-09-21

**Authors:** Sudhanshu Kumar, Arun Chakraborty, Raghvendra Chandrakar, Abhishek Kumar, Biplab Sadhukhan, Riyanka Roy Chowdhury

**Affiliations:** 1 Centre for Ocean, River, Atmosphere and Land Sciences (CORAL), Indian Institute of Technology Kharagpur, Kharagpur, 721302 India; 2 Department of Crop, Soil, and Environmental Sciences, Auburn University, Auburn, AL USA; 3 Ocean Process Analysis Laboratory, University of New Hampshire, Durham, NH USA

Correction to: *Scientific Reports* https://doi.org/10.1038/s41598-023-39884-y, published online 30 August 2023

The original version of this Article omitted an affiliation for Sudhanshu Kumar. Their correct affiliations are listed below.

Centre for Ocean, River, Atmosphere and Land Sciences (CORAL), Indian Institute of Technology Kharagpur, Kharagpur, 721302, India.

Department of Crop, Soil, and Environmental Sciences, Auburn University, Auburn, AL, USA.

The original Article has been corrected.

