# Supplementary material for: Analysis of marine heatwaves over the Bay of Bengal during 1982–2021
Source: Sci Rep. 2023 Aug 30;13:14235. doi: 10.1038/s41598-023-39884-y (PMC10468509; doi:10.1038/s41598-023-39884-y)
Supplement: Supplementary file 1 — Supplementary Figures. [file 41598_2023_39884_MOESM1_ESM.docx]

**Supplementary Figures**


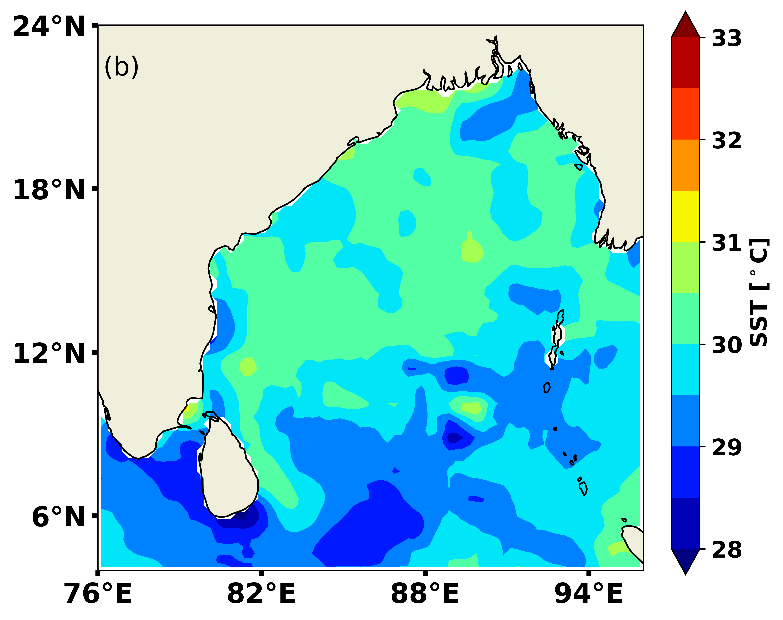

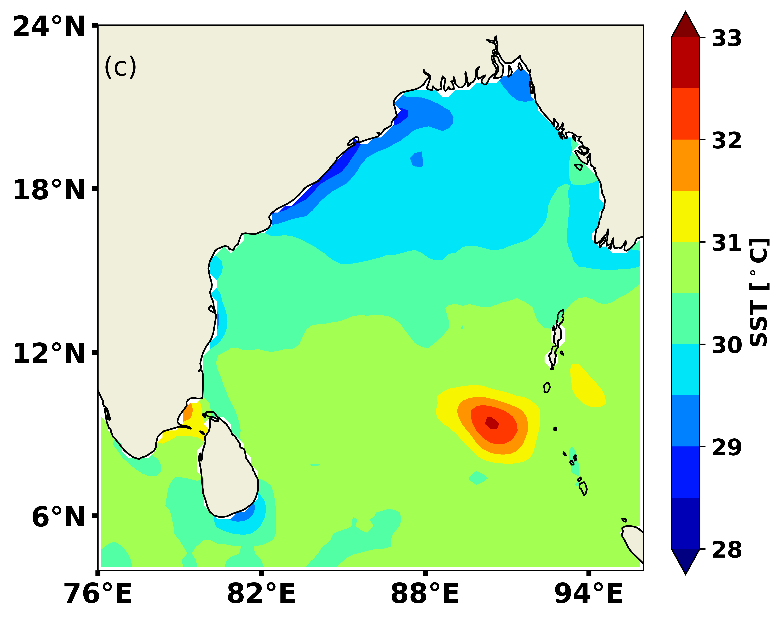

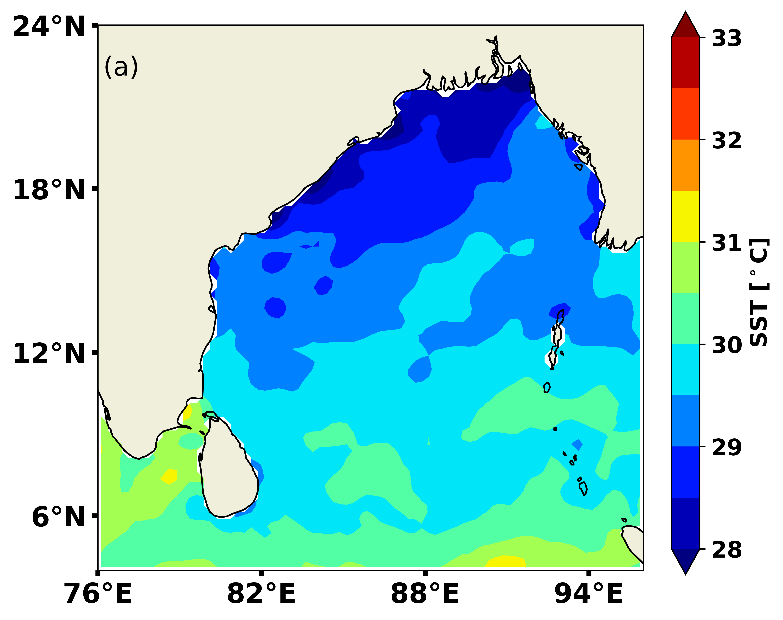

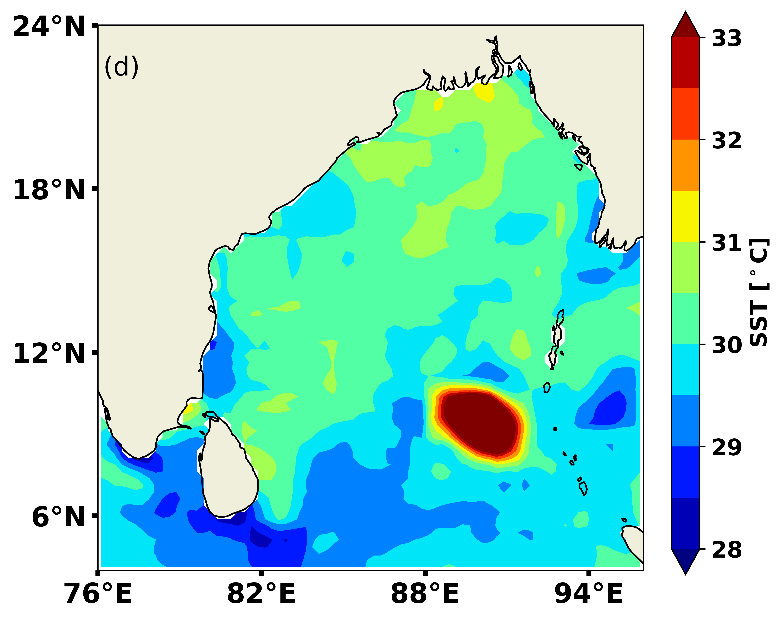


**Figure S1:** Spatial variation of SST (°C) over the Bay of Bengal on the (a) starting day of the most intense MHW event, (b) ending day of the most intense MHW event, (c) mean of all days of most intense MHW event, and (d) the day of maximum intensity of most intense MHW event.


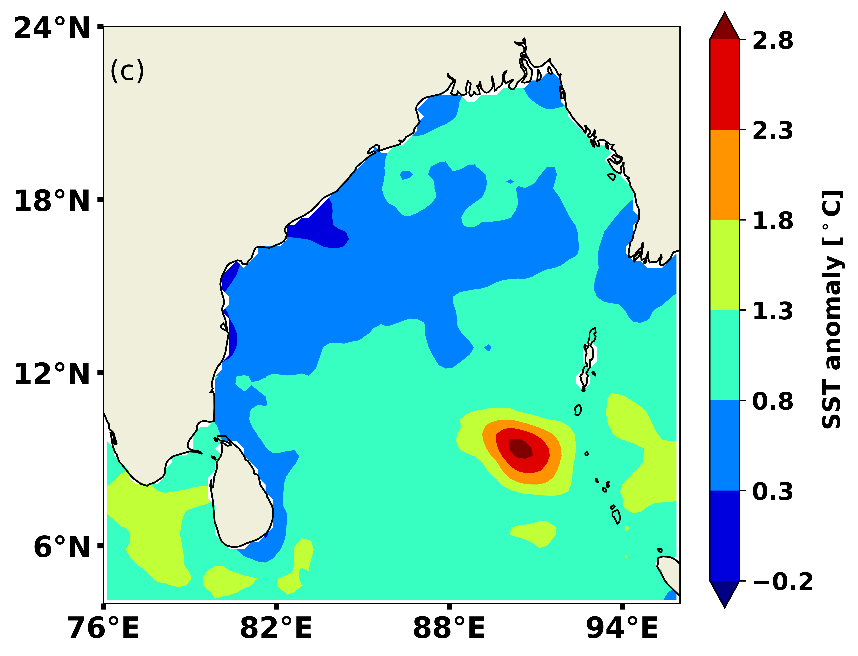

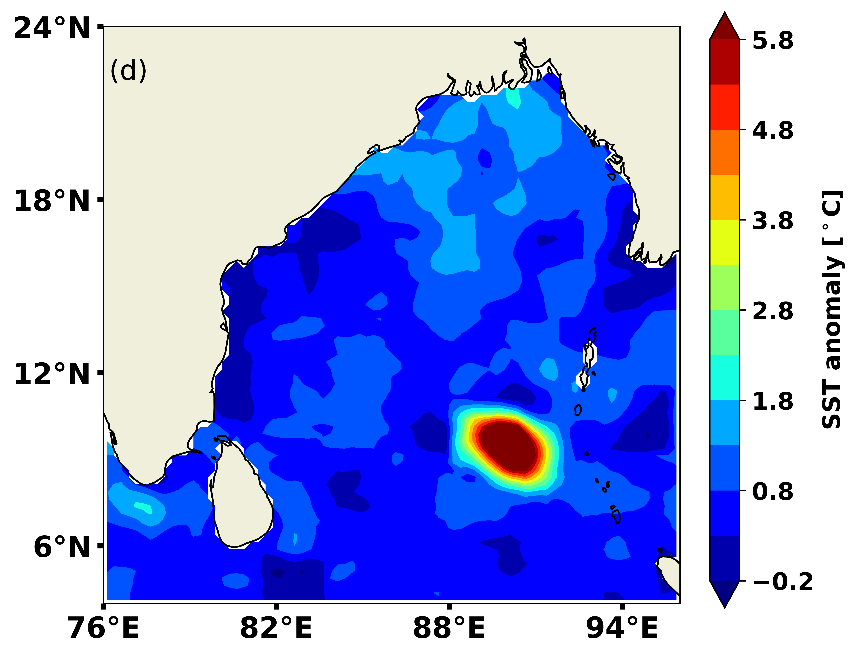

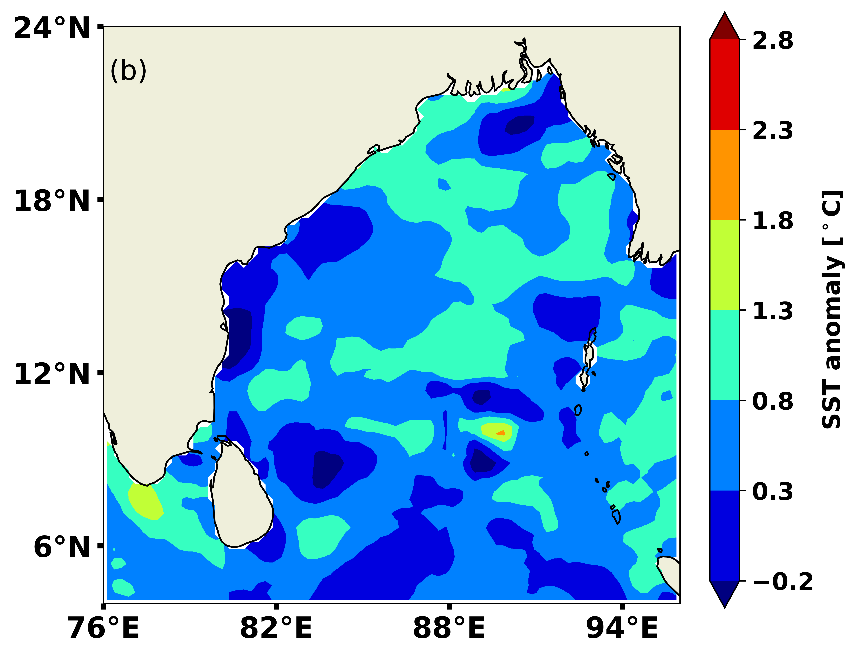

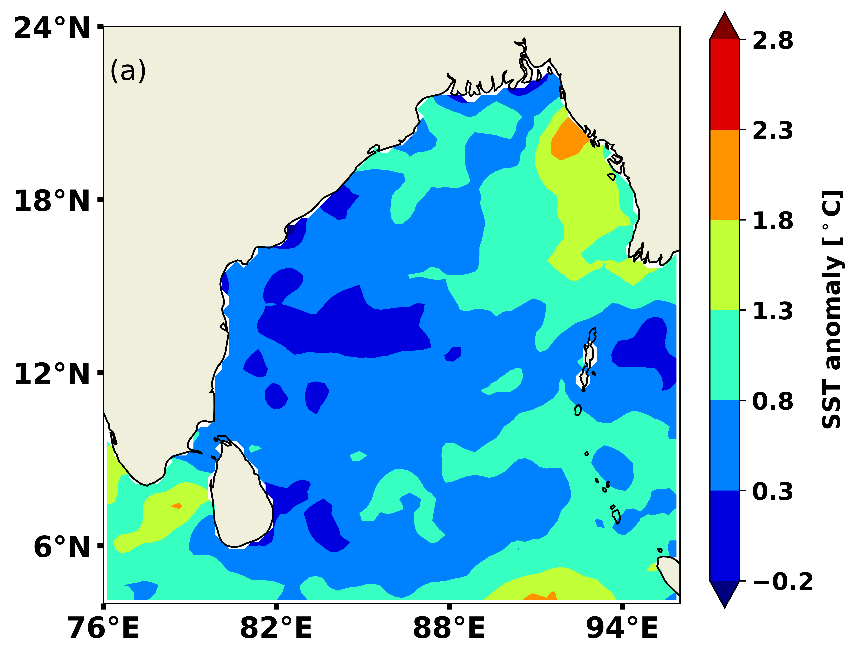


**Figure S2:** Spatial variation of SST anomaly (°C) over the Bay of Bengal on the (a) starting day of the most intense MHW event, (b) ending day of the most intense MHW event, (c) mean of all days of most intense MHW event, and (d) the day of maximum intensity of most intense MHW event.
